# Placenta, Pericarp, and Seeds of Tabasco Chili Pepper Fruits Show a Contrasting Diversity of Bioactive Metabolites

**DOI:** 10.3390/metabo9100206

**Published:** 2019-09-28

**Authors:** Felipe Cervantes-Hernández, Paul Alcalá-González, Octavio Martínez, José Juan Ordaz-Ortiz

**Affiliations:** Unidad de Genómica Avanzada, Centro de Investigación y de Estudios Avanzados del Instituto Politécnico Nacional (CINVESTAV), Km. 9.6, Libramiento Norte Carretera Irapuato-León, Irapuato, Gto. 36824, Mexico; felipe.cervantes@cinvestav.mx (F.C.-H.); paulpascual92@hotmail.com (P.A.-G.); octavio.martinez@cinvestav.mx (O.M.)

**Keywords:** *Capsicum frutescens* L., non-targeted metabolomics, secondary metabolism, Liquid Chromatography coupled to Mass Spectrometry (LC-MS)

## Abstract

Chili pepper (*Capsicum* spp.) is one of the most important horticultural crops worldwide, and its unique organoleptic properties and health benefits have been established for centuries. However, there is little knowledge about how metabolites are distributed throughout fruit parts. This work focuses on the use of liquid chromatography coupled with high resolution mass spectrometry (UHPLC-ESI-HRMS) to estimate the global metabolite profiles of the pericarp, placenta, and seeds of Tabasco pepper fruits (*Capsicum frutescens* L.) at the red mature stage of ripening. Our main results putatively identified 60 differential compounds between these tissues and seeds. Firstly, we found that pericarp has a higher content of glycosides, showing on average a fold change of 5 and a fold change of 14 for terpenoids when compared with other parts of the fruit. While placenta was the richest tissue in capsaicinoid-related compounds, alkaloids, and tocopherols, with a 35, 3, and 7 fold change, respectively. However, the seeds were richer in fatty acids and saponins with fold changes of 86 and 224, respectively. Therefore, our study demonstrates that a non-targeted metabolomic approach may help to improve our understanding of unexplored areas of plant metabolism and also may be the starting point for a detailed analysis in complex plant parts, such as fruits.

## 1. Introduction

Chili pepper (*Capsicum* spp.) is one of the most important crops worldwide. It is used as a main ingredient for many dishes in different cultures, such as Asian, Latin-American, and Mediterranean cultures, due to its organoleptic properties [1]. There are 40 accepted chili species but only five are considered domesticated: *C. annuum*, *C. chinense* Jacq, *C. frutescens*, *C. baccatum*, and *C. pubescens* [2]. In 2017, 36 million tons of chili peppers were produced globally, with Mexico being the second largest producer [3]. Wild pepper populations of Tabasco pepper (*C. frutescens* L.) and *C. annuum* can be found in some states of Mexico, increasing the relevance for collecting and characterizing these species as resources for the breeding of cultivated peppers [4,5]. Previous studies have extensively described metabolite diversity in *C. annuum* [6,7] but very little is known on *C. chinense* and *C. frutescens*. Most of these studies have undertaken a targeted approach, where the main focus has been to quantitate for specific metabolites, such as capsiate, dihydrocapsiate, capsaicin, dihydrocapsaicin, carotenoids, fatty acids, and amino acids [8,9]. To date, there are no studies with a comprehensive global profiling of tissue specific in *C. frutescens* and *C. chinense*.

*Capsicum* species are known to be rich in compounds such as capsaicinoids, capsinoids, carotenoids, flavonoids, vitamins, essential oils, and other phytochemicals, which provide a unique taste, aromatic properties, and health benefits [10,11,12,13,14]. Capsaicinoids consist mainly of two congeners, capsaicin and dihydrocapsaicin. Capsinoids also have two major analogues, capsiate and dihydrocapsiate. However, there are structural differences: Capsaicinoids are fatty acid amides linked with vanillylamine, whereas capsinoids are fatty acid esters linked to vanillyl alcohol [15,16]. Several compounds identified in the *Capsicum* species have been studied, because the medicinal potential examples of these compounds are icariside E5, capsosides, and capsianosides [17]; hydroxycinnamic derivatives, O-glycosides of quercetin, luteolin, and chrysoeriol [7]. The reported medicinal benefits are related to areas, such as anti-inflammatory [18], anti-cancer [19], anti-microbial, antioxidant properties [1,20,21], and those with weight-loss properties [22]. In addition, some epidemiological studies of a number of these antioxidants reported that they possess anti-atherosclerotic, antitumor, antimutagenic or anticarcinogenic activity [23,24,25]. It may be that these properties have the ability to help us to address the identification, isolation, and production of nutraceutical compounds or new natural medicinal compounds.

Nevertheless, the location and relative abundance of these metabolites and their precursors in different parts of the chili pepper fruit, such as the pericarp, placenta and seed, remain unclear. It is known that some compounds are synthesized and accumulated into specific tissues in the *Capsicum* genus [1,26]; for example, capsaicin is synthesized mainly in the placenta [27,28], while anthocyanins are described as being accumulated in pericarp during fruit development [29]. Materska, reported a placental and pericarp comparison in chili pepper fruit, where placenta was the richest in flavonoids, while the pericarp presented a larger diversity in glycosylated compounds. Despite this, little is known about the tissue specific spatial-temporal location of other classes of compounds and products of the secondary metabolism in *Capsicum* fruits [30]. Investigating the metabolic diversity on fruit tissues is essential in order to gain a comprehensive understanding of the function of specific parts of the fruit at the fundamental level. Consequently, this will enable the possible exploitation of natural products either for pharmaceutical or food products. In the past, these have been done with histochemical methods, by staining tissues sections with various chemical to reveal the presence of specific compounds either by visual or microscope inspection [31].

Metabolomics is defined as the comprehensive analysis of all low molecular weight organic compounds (<1500 Da) in a biological system [32]. Mass spectrometry has become the most widely applied platform for metabolomics, due to the wide range of molecules that can be analyzed on a single run [33]. Global profiling or non-targeted mass spectrometry-based metabolomics have gained importance in the study of crop species and have been applied to investigate potato, tomato, rice, wheat, strawberry, cucumber, and tobacco [34,35,36,37]. In the field of plant metabolomics, liquid chromatography coupled with electrospray ionization high resolution mass spectrometry (UPLC-ESI-HRMS) has emerged as the technique of choice for the putative identification of metabolites in complex matrices. This technique has been widely used, due to its sensitivity, selectivity, and analysis capability [38,39]. Nevertheless, metabolite identification for unknown compounds still remains a big challenge to overcome. In that respect, the recommendations by the Metabolomics Standards Initiative (MSI) recognize five different levels for metabolite confidence annotations. Level 0 requires the full compound 3D structure and stereochemistry information. Levels which are more common include: Level 1 identifications need a confirmation by two orthogonal parameters such as retention time and MS/MS spectrum, normally with match reference standards; and Level 2 requires at least two orthogonal pieces of information, including evidence that excludes all other candidates. Data for Level 2 should describe probable structure and be matched to literature data or databases by diagnostic evidence [40].

Combining existing bioinformatic tools with high resolution mass spectrometry data can reveal unclear relationships of metabolites and their possible function at a spatial-temporal distribution level. As a first attempt to construct the chili fruit metabolome, we produced a hand curated dataset, that contains 60 putative identified metabolites, which include alkaloids and terpenoids that are unreported in *Capsicum*, with significant differences and relative abundances between three sections (pericarp, placenta, and seed) of Tabasco pepper fruit at the mature red stage, using an UHPLC-QTOF-HRMS platform, combined with the use of Progenesis QI for small molecules, as a tool for the pre-identification of unknown metabolites. Our results underline the global metabolic differences in complexity, mainly based on the secondary metabolism of these fruit parts.

## 2. Results

### 2.1. Non-Targeted Metabolomic Analysis

**Figure 1:**
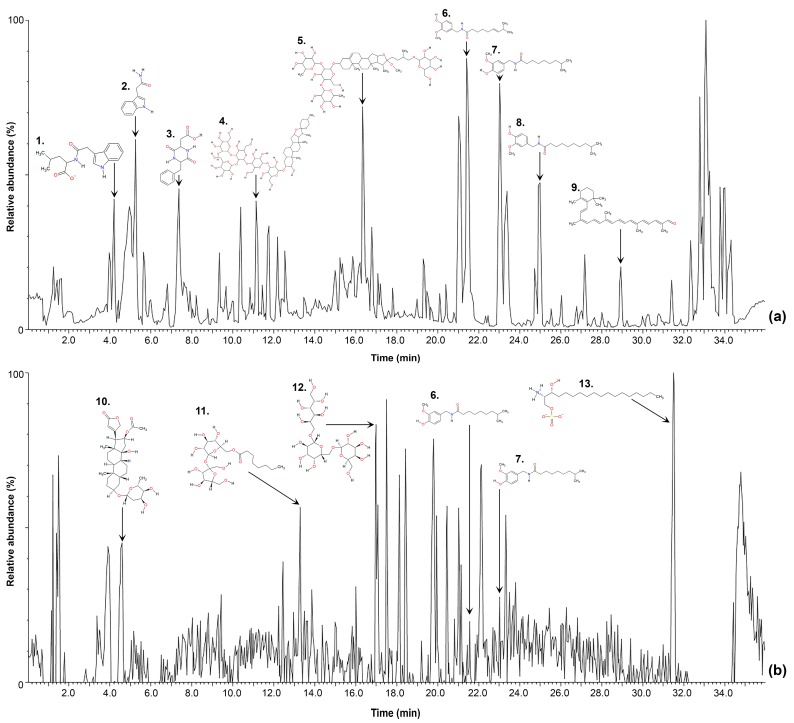
(**a**) Base peak intensity chromatographic profile of placenta tissue from chili pepper fruit on a Charged Surface Hybrid (CSH) C18 column obtained with Electrospray Ionization (ESI) positive mode on a mass range from 100 to 1500. 1. 2-[(1H-Indol-3-ylacetyl) amino]-4-methylpentanoate; 2. Indole-3-acetamide; 3. L-cis-Cyclo (aspartylphenylalanyl); 4. Capsicosin; 5. Yamogenintetroside B; 6. Capsaicin; 7. Dihydrocapsaicin; 8. Homodihydrocapsaicin; 9. β-Carotinal. (**b**) Base peak intensity chromatographic profile of placenta tissue from chili pepper fruit on a CSH C18 column obtained with ESI negative mode. 10. Oleandrigenin monodigitoxoside; 11. β-d-fructofuranosyl 6-O-octanoyl-α-d-glucopyranoside; 12. β-(1->6)-galactotriitol; 13. (2S,3R)-2-Azaniumyl-3-hydroxyoctadecyl phosphate.

Our data for two tissues and the seed of Tabasco chili fruit comprised a total of 87 files or chromatograms per ionization mode (positive and negative polarities) as shown in Figure 1 and were uploaded into Progenesis QI. The dataset was first aligned (retention time): Each chromatogram was aligned against each other and automatically compared to a reference profile selected by the software that contained the highest number of features (potential compounds). Then, peak picking was performed by default parameters. A total of 1980 features were detected in the aqueous phase and 1481 were detected in the diethyl ether extract, for both ionization modes. Figure 1 shows a typical chromatographic profile in positive and negative ionization mode of placenta tissue, displaying some representative compounds of chili pepper fruit.

**Figure 2:**
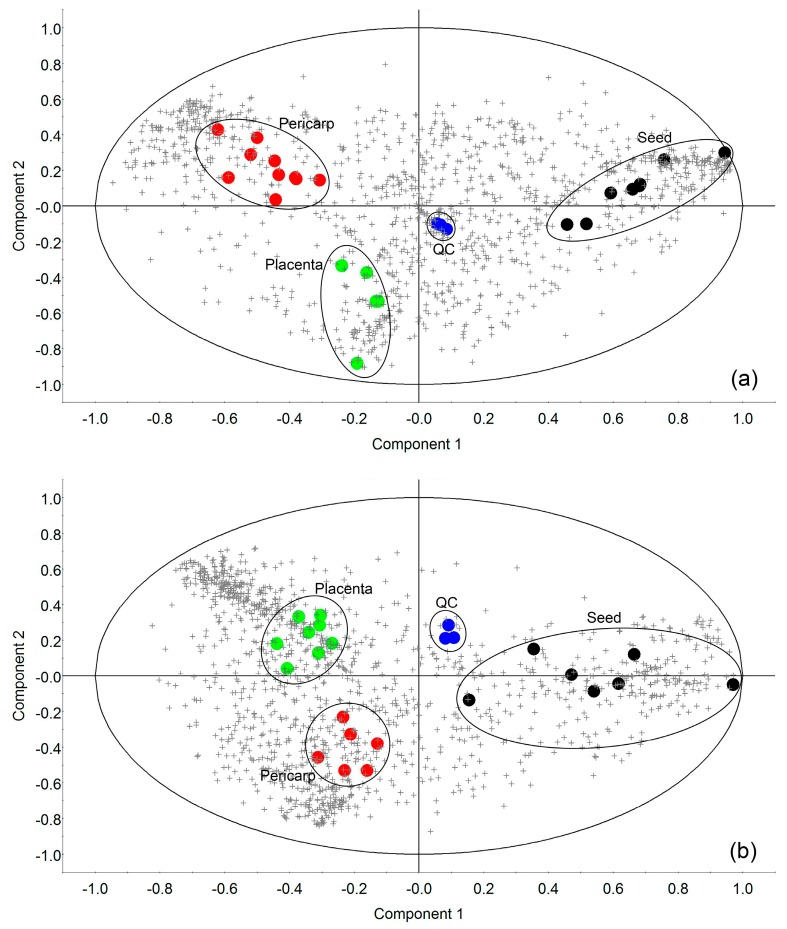
(**a**) Principal Component Analysis (PCA) Bi-plot of loadings (features: crosses) obtained in ESI positive mode and scores (samples: colored circles) extracted with methanol:water phase (component 1:33.10%; component 2: 15.72%; loadings = 1294 features; n = 26); (**b**) PCA Bi-plot of loadings (features: crosses) and scores (samples: colored circles) extracted with diethyl ether phase (component 1:37.15%; component 2: 15.14%; loadings = 1391 features; n = 24).

Distribution of the features between parts was compared using principal component analysis (PCA) of loading and score plots describing a significant grouping by part for both extraction phases (Figure 2). In addition, quality controls (QC samples) were also considered, as shown in Figure 2. The QC samples cluster tightly in comparison to the total variance in the projection, suggesting a dataset deemed to be of high quality. Table 1 and Table 2 describe loadings that mostly contribute to principal components for each extraction. Putative organic compounds catalogued as saponins (SPNS), such as Tuberoside J, Matesaponin 5, and Asparagoside B significantly contributed to component 1 in the aqueous phase, while other important features for component 2 belong to flavonoid class (FLV) and SPNS compounds. Furthermore, both components in the organic phase were mainly composed of glycerolipids (GL) and terpenoids (TER), as well as a putative carotenoid, (5cis,5′cis,9cis,11′cis)-1,2,7,7′,8,8′-Hexahydro-1,2-epoxy-ψ, ψ-carotene were important for contribution of component 1 in organic phase.

### 2.2. Level 1 and 2 Metabolomic Identification Analysis

We putatively identified approximately 270 compounds and classified them in 52 compound classes (Table S1). Putative identifications were taken into consideration with a high match score (>90%). Terpenoids, fatty acids, and glycosylated compounds were the most abundant groups. As was predicted, different capsaicinoid compounds were also detected with a high match score. Alkaloids, carotenoids, saponins, and phenolic compounds were also detected in our study.

**Figure 3:**
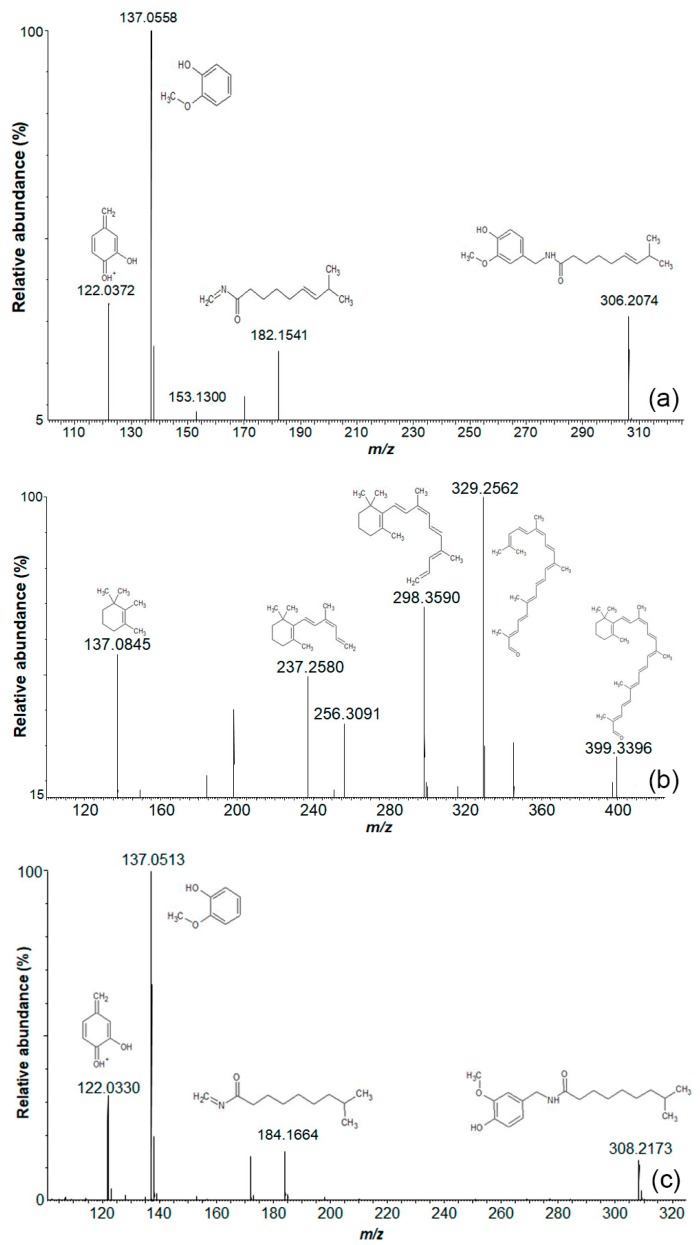
Mass spectrum of most common compounds in Tabasco chili pepper using Ultra High Pressure Liquid Chromatography MSMS Quadrupole Time of Flight (UHPLC-MS^2^ Q-TOF; collision energy ramp: 20–40 eV) ESI positive ionization mode of placenta tissue, as putatively identified by Progenesis QI for small molecules. (**a**) Capsaicin; (**b**) β-carotinal; and (**c**) dihydrocapsaicin.

Capsaicin and dihydrocapsaicin were validated by matching their retention times and MS/MS spectra with those of the analytical standard (Level 1 identification). Furthermore, commonly reported compounds, such as carotenoids and capsaicin related compounds, were detected and putatively identified in the same manner in our samples (Figure 3).

### 2.3. Different Metabolomic Profiles in Capsicum Sections

**Figure 4:**
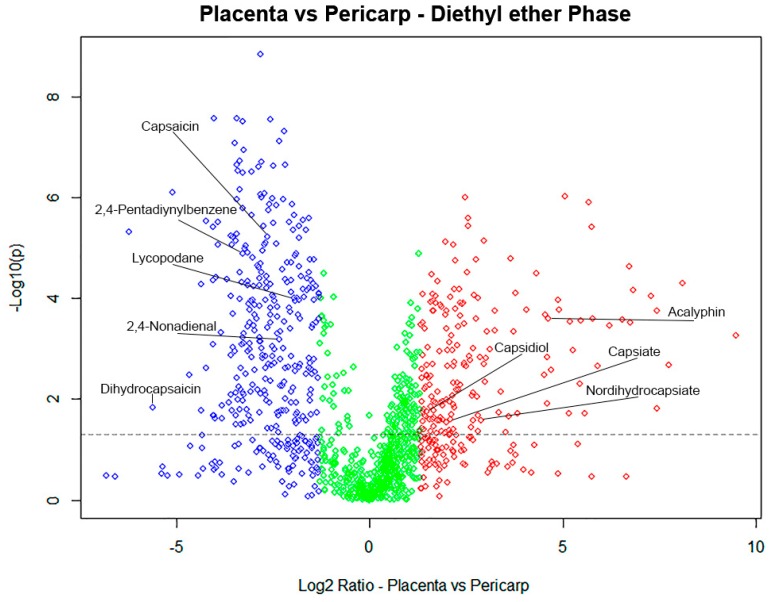
Volcano plot comparison of relative abundance between tissues of 1394 features in ESI positive ionization mode: Placenta (left), and pericarp (right), unchanged (green); one-way ANOVA *p* = 0.05 (dotted line) Y axis: p value, X axis: fold change.

Based on results from volcano plot comparisons, we found a total of 60 putative compounds with significant differences between the parts of the Tabasco pepper fruit. Figure 4 shows the distribution of features between placenta and pericarp tissues. As was expected [28], capsaicinoids were more abundant in placenta than pericarp. In contrast, pericarp was richer in glycosylated compounds and terpenoids such as acalyphin, capsiate, and capsidiol. Some significant ions remain unknown that still need to be identified.

**Figure 5:**
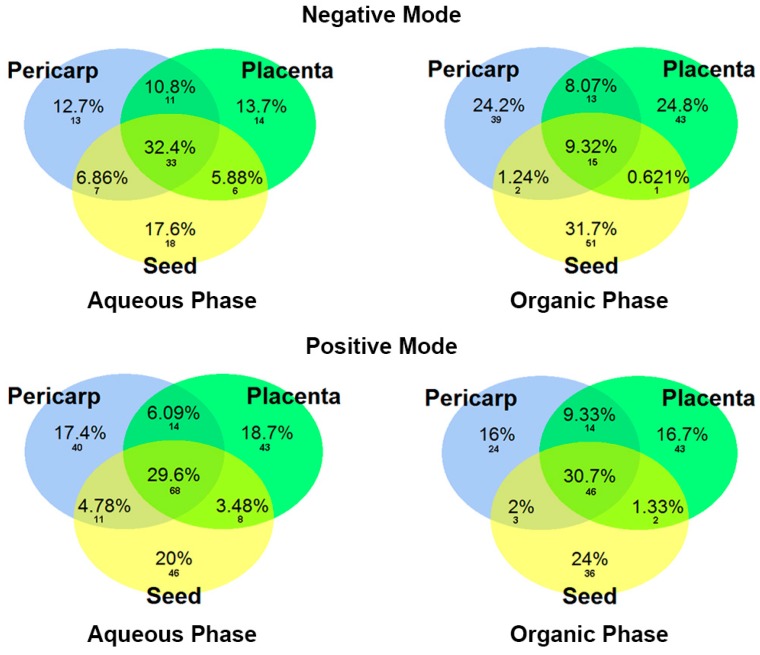
Venn diagrams of the complete dataset of putative metabolites in different fruit parts at the red mature stage of Tabasco chili pepper (*C. frutescens*); labels are in percent and number of metabolites.

After feature screening and putative identification, Venn diagrams were generated (Figure 5) to show similarities and differences between fruit parts, according to the fold change values obtained in Table S1. Noticeably, around 30% of the complete dataset of identification was shared by all three fruit parts in almost all the extraction solvents, the exception being for the organic phase in the negative ionization mode, which only share 9.32% similarities. As can be seen in the Venn diagrams, a greater number of putative metabolites were identified in the positive ionization mode. Those easily detected in the positive ionization mode were the compound classes alkaloids, carotenoids, fatty acids, glycosylated compounds, terpenoids, and saponins; while in the negative ionization mode amino acid-derivate compounds, sphingolipids, and phospholipids were detected. 

Listed in Table 3 is the putative identification that presents a differential abundance between fruit parts, including the fragmentation pattern (MS^2^) and adducts for putative annotation. Fold change values are shown in Table S1.

## 3. Discussion

The global metabolic comparison between the tissues and seeds of *C. frutescens* showed several feature differences between the pericarp, placenta, and seed. The Level 1 and 2 confidence metabolite annotations allowed us to assign a putative identification to these ions. Around 30% of metabolites were shared between all three parts. Compounds related to the primary metabolism showed few significant differences, they included amino acid related compounds, fatty acids, and phospholipids. As shown in the Venn diagram (Figure 5) and Table S1, placenta and pericarp have the biggest compound class diversity. Significantly, the seeds presented a higher number of putative identifications, and these were primarily saponins, terpenes, and fatty acids.

Pericarp compound classes were mainly composed of glycosylated compounds and terpenoids. Complementary to these findings, Materska demonstrated that chili pepper pericarp is abundant in glycosylated compounds [30]. Likewise, terpenoids were distributed in the whole fruit but pericarp showed a slightly higher proportion of them. These compounds are highly abundant in spices and herbs and give a wide range on the aroma and flavor spectrum [1]. Similarly, terpenoids have been described as showing antibiotic properties [41] and have been used in fragrances [42].

Placental tissue showed a large number of previously reported compounds with bioactivity, mainly capsaicin- and capsinoids-related compounds. In addition, alkaloids and tocopherols were present, a fact that is in agreement with current literature [28,30]. Found to be abundant in this fruit compartment were 6-O-acetylaustroinulin (terpenoid) and Myrciacitrin V (flavonoid) and they have not been reported in the *Capsicum* fruit.

The compounds found in chili seeds were predominantly fatty acids (3,16-Dihydroxypalmitate, Sterculynic acid) and saponins (Capsicoside A, Eleutheroside L and Tragopogonsaponin F) where they function as reserve nutrients for embryo development and propagation [43]. Moreover, seeds showed the presence of terpenes (Ursolic acid 3-[glucosyl-(1->4)-xyloside]) which are known to function as a natural promoter of predation and, as a consequence, a seed disperser [44]. Ritota et al. (2010) reported an abundance of fatty acids in sweet pepper species by nuclear magnetic resonance spectroscopy, in which polyunsaturated fatty acids were easily detected and pre-identified [6].

Our results were consistent with previously reported findings regarding the large diversity of secondary metabolites in fruits of *Capsicum* species and the non-targeted metabolomics profiling of Solanaceae [10,26,39,45,46,47,48]. Furthermore, new compounds, such as Myrciacitrin V, Feruloyl-β-sitosterol, 6-O-acetylaustroinulin and others, were putatively annotated as statistically significant in specific fruit parts.

Capsaicin, Dihydrocapsaicin, and capsaicinoids derivatives mainly accumulate in the placenta, as previously reported [27,49]. This class of compounds represents the most described and abundant metabolite in this genus and are predominantly known as being responsible for the pungency. Different bioactivity assays have been developed, demonstrating properties of capsaicinoids over different cell lines and metabolism, including as an analgesic and for weight-loss [22,50]. Large abundance of capsaicinoids in the chili fruit placenta was proposed by Tewksbury and Nabhan (2001), who suggest that capsaicin selectively discourages vertebrate predators (capsaicin has been found to repel or poison mammals) without deterring more effective and important seed dispersers, such as birds. [51].

A variety of new compounds in *Capsicum* genus, also reported in different species, were detected in pericarp, including isopimaric acid [41], which is a terpenoid with bioactive properties. Additionally, other compounds such as Pedalitin [52], Xi-8-acetonyldihydrosanguinarine [53], Pratenol B [54], Uralenneoside [55] were detected in pericarp and have been previously reported as bioactive compounds. Quercetin 3-(6″-malonyl-glucoside) is an anthocyanin-related compound that has not been reported in pepper fruit, but this compound class is well known to be localized mainly in pericarp [56,57], due to its involvement as a protection system against solar damage in plants and to attract potential pollinators [29].

New putative compounds in placental tissue, such as Lycopodane [58], 2,4-Pentadiynylbenzene [59], Myrciacitrin V [60], Cinncassiol C [61], and 6-O-acetylaustroinulin [62] have been reported as bioactive compounds, supporting the nutraceutical properties of chili pepper against metabolic disorders. 

The existence of terpenes in seeds may result in different aromas that have been shown to firstly attract birds to mature fruits during the day [63] and secondly, to promote the dispersal of seeds. This function supports the ecological relationship between birds and chili pepper fruits, attracting the most beneficial vertebrate predators [51].

In summary, the non-targeted LC-MS metabolomics method that was developed in this study is shown to be a powerful tool for the putative identification of tissue-specific secondary metabolites at the red mature stage of chili pepper fruit. The use of databases available online gave rise to a faster comprehensive elucidation of global characteristics of a complex matrix than more traditional phytochemical studies. Nutraceutical, aroma, flavor, and new compounds that have not been reported before were putatively identified and related to pericarp, placenta or seeds of *C. frutescens*. As presented here, some of these compounds have been reported with bioactivity properties, supporting empirical properties of pepper fruit that have been known for centuries. The procedure developed here will be utilized for further studies in our laboratory, including to enable the exploration of comparisons between wild cultivars of chili pepper fruit with their cultivated counterparts and for the further understanding of secondary metabolism in this crop. We recommend that complementary analysis should be carried out to confirm structural elucidation. In addition, compound isolation and bioactivity properties should be considered in future studies.

## 4. Materials and Methods 

### 4.1. Plant Material and Dissection of Tissues and Seed

Seeds of Tabasco pepper (*C. frutescens* L.) were treated with 3% hypochlorite solution. Plants were grown in optimum conditions (30–32 °C), at greenhouse facilities between June and September of 2016. Fruits from different plants were collected at 60 DPA (red ripe stage), washed with deionized water and immediately frozen with liquid nitrogen and stored at −80 °C until dissection and analysis.

Five biological replicates (plants) were considered for the experiment and three fruits per plant were collected. Each fruit was first placed into dry ice to facilitate hand dissection into pericarp, placenta, and seed using a sterile scalpel. All fruit parts were ground using a ball mill (Retsch MM301) under cold conditions and applying liquid nitrogen.

### 4.2. Chemicals, Reagents, and Standards

All chemicals and reagents were purchased from AccesoLab S.A. de C.V. (Mexico, Mexico). Capsaicin and dihydrocapsaicin analytical standards, formic acid, methanol, acetonitrile were HPLC grade and purchased from Sigma–Aldrich (Mexico, Mexico). 

### 4.3. Sample Extraction and UHPLC-MS Analysis

For metabolite extraction, the method employed was adapted from Matyash [64] as follows: Methanol, 1.5 mL, was added to 100 mg of sample in a test tube and vortexed for 1 min, then, 5 mL of diethyl ether was added. The mixture was incubated with gentle stirring for one hour at room temperature. Next, 1.5 mL of ultra-pure water (18 Ω, milli-Q system) was added and mixed vigorously for a further minute then kept at room temperature for 10 min to allow phase separation. After that, the sample was centrifuged at 1000 × g for 10 min. Aqueous and organic layers were recovered separately and vacuum dried (miVac®, Genevac) at 30 °C for 30 min and finally kept at −80 °C until further analysis.

Three quality control (QC) samples were prepared to account for instrument drift and system calibration during analysis in UHPLC-QTOF-HRMS; each QC sample was prepared by mixing homogenously all sample extracts into a new single vial, in both separated phases containing polar and non-polar compounds. QC samples were distributed at the beginning, middle, and end of the injection run list. Analytical standards of capsaicin and dihydrocapsaicin were injected under the same conditions as samples. Extraction blanks were also considered during the experiment.

For LC-MS analysis, all samples (including QC, analytical standards and blank extraction) were resuspended in 1 mL of acetonitrile/ultra-pure water 50:50 (*v/v*) and filtered through a membrane of 0.2 µm (PTFE, Agilent Technologies, Santa Clara, USA). Samples were injected according to a randomized list order on an UPLC® (Acquity class I, Waters, Milford, CA, USA) coupled with an orthogonal QTOF (SYNAPT G1 HDMS, Waters, Milford, CA, USA) mass spectrometer. Chromatographic separation was achieved on a reversed phase CSH C18 column (2.1 mm × 150 mm, 1.7 µm, Waters, Milford, USA) maintained at 30 °C during chromatographic separation. Auto-sampling of 10 µL per sample was injected. Compounds were eluted using ultra-pure water with 0.1% (*v/v*) formic acid (solvent A) and acetonitrile with 0.1% (*v/v*) formic acid (solvent B) with a flow rate of 0.3 mL/min with the following gradient program: From 0.5 to 30 min, 1–75% B; 30 to 31 min, 75% B; 31 to 31.5 min, 75–100% B; 31.5 to 34.5, 100% B; 34.5 to 34.6, 100-1% B; 34.6 to 36 min, 1% B. The mass spectrometer mass range was set from 50 to 1500 Da. Both ionization modes were injected separated. For negative electrospray ionization (ESI) mode, the conditions were set as follows: Capillary voltage 2 kV; cone voltage 40 V; source temperature 150 °C; cone gas flow 20 L/h; desolvation temperature 350 °C; desolvation gas flow 600 L/h. For the positive ESI mode: Capillary voltage 3 kV; cone voltage 40 V; source temperature 130 °C; desolvation temperature 350 °C; desolvation gas flow 700 L/h. Leucine-Enkephalin (2 ng/mL) was infused as LockSpray reference internal mass calibrant at a flow rate of 5 µL/min and its signal was monitored every 10 s. The data format was collected in a continuum mode with a MS scan time of 1.5 s. In both the positive and negative ionization mode, data were acquired in MS^E^ experiments; using Argon as the collision gas with a collision energy in the trap region of 6 eV (Function 1, low energy) and ranged from 20–40 eV (Function 2, high voltage).

### 4.4. Data Analysis

Raw data was imported to Progenesis QI for small molecules software (Non-Linear Dynamics, Waters, Milford, MA, USA) for automatic alignment, normalization, deconvolution, and compound pre-identification over all samples separating the aqueous and organic phases. The RT range was limited from 0.5 to 35 min for pre-identification method. Pre-identification was performed using Chemspider Databases (PlantCyc, Plant Metabolic Network, KEGG, HMDB and ChEBI) and with an in-house database with a minimum match of 90% for precursor ions, MS/MS data and isotope distribution was included for increasing match score values. Statistics and graphics were performed using EZinfo 3.0 (Waters, Milford, MA, USA) and R (3.3.3v, Vienna, Austria) [65] software. Compounds were grouped according to their compound classes. The resulting data was first mean centered and scaled to Pareto and then submitted to a principal component analysis (PCA) using the first three components. Results were analyzed using one-way ANOVA and q-values were established using the false discovery rate (FDR < 0.01) to correct multiple comparisons by the Benjamini–Hochberg procedure [66].

## Figures and Tables

**Table 1:** Loadings most contributing to principal components for the aqueous phase.

Putative Identification	Class	PC1
Tuberoside J	SPNS	0.2012
Asparagoside B	SPNS	0.1939
Matesaponin 5	SPNS	0.1871
Oleanolic acid 3-O-[O-β-d-glucopyranosyl-(1->4)-O-β-d-glucopyranosyl-(1->3)-O-α-l-rhamnopyranosyl- (1->2)-α -l-arabinopyranoside]	SPNS	0.1822
Capsicosin	SPNS	0.1650
		PC2
(3″-Apiosyl-6″-malonyl) astragalin	FLV	0.1401
Pratenol B	BZD	0.0881
Asparagoside B	SPNS	0.0750
Matesaponin 5	SPNS	0.0673
Kaempferol 3-xylosylglucoside	FLV	0.0658

PC1: Principal Component 1; PC2: Principal Component 2; BZD: benzoyl derivate; FLV: flavonoid; SPNS: saponin.

**Table 2:** Loading most contributing to principal components for the organic phase.

Putative Identification	Class	PC1
Abietane	TER	0.1450
(5cis,5′cis,9cis,11′cis)-1,2,7,7′,8,8′-Hexahydro-1,2-epoxy-ψ, ψ-carotene	CARO	0.1429
Lycoperoside D	SPNS	0.1324
Phyllohydroquinone	TER	0.1302
2-Caprylooleomyristin	GL	−0.0796
		PC2
α,α′-Trehalose 6-mycolate	GL	0.1505
2-Caprylooleomyristin	GL	0.1226
MG(14:0/0:0/0:0)	GL	−0.1284
Uralenneoside	BZD	−0.0757
Abietane	TER	0.0719

PC1: Principal Component 1; PC2: principal Component 2; BZD: benzoyl derivate; Caro: carotenoid; GL: Glycerolipids; SPNS: saponin; TER: terpenoid.

**Table 3:** Differential putative identifications in parts of Tabasco pepper fruit by UHPLC-MS^2^ in both ESI modes.

Compound Name	Formula	Class	Adduct	Precursor (*m/z*)	Fragments (*m/z*)
α-campholenaldehyde	C_10_H_16_O	TER	[M + H − H_2_O] +	135.1180	109.1021(3.7)
Jasmolone	C_11_H_16_O_2_	JASM	[M + H − 2H_2_O] +	145.1027	133.1026(4.0), 121.1024(3.3), 107.0864(3.5)
2,4-Pentadiynylbenzene	C_11_H_8_	BZD	[2M + NH4] +	298.1669	177.0684(2.8), 145.0399(1.6), 117.0428(1.9)
Uralenneoside	C_12_H_14_O_8_	BZD	[M + H] +	287.0755	287.0741(63.6), 285.0609(0.4), 257.0637(2.6), 203.0493(0.5), 153.0300(1.45), 135.0542(0.1)
Synephrine acetonide	C_12_H_17_NO_2_	BZD	[2M + FA − H] -	459.2565	208.2805(8.9)
Cuscohygrine	C_13_H_24_N_2_O	AK	[M + H] +	225.1977	197.1340(5.5), 183.1184 (5.6)
Acalyphin	C_14_H_20_N_2_O_9_	GC	[M + Na] +	383.1044	325.0952(1.3), 299.0774(4.6), 165.0311(0.3)
Pratenol B	C_15_H_12_O_7_	BZD	[M + H − H_2_O] +	287.0546	153.0195(2.8), 131.0512 (2.3)
Lycopodane	C_15_H_25_N	AK	[M + H − 2H_2_O] +	220.3782	184.1841(5.8)
Pedalitin	C_16_H_12_O_7_	FLV	[M + H − H_2_O] +	299.0570	299.0568(7.3), 165.0197(0.5)
Nordihydrocapsaicin	C_17_H_27_NO_3_	CAPS	[M+H] +	294.2055	285.2240(3.6), 257.2282(2.8), 189.1653(3.9)
Nerolidyl acetate	C_17_H_28_O_2_	TER	[M + H − 2H_2_O] +	229.1966	161.134 (12.7)
Capsaicin	C_18_H_27_NO_3_	CAPS	[M + H] +	306.2075	182.1559(0.2), 137.0605(15.4), 122.0371(5.8)
Dihydrocapsaicin	C_18_H_29_NO_3_	CAPS	[M + H] +	308.2240	9137.061 (5.5)
Artocarbene	C_19_H_18_O_4_	PPN	[M + H] +	311.1301	175.0771(2.5), 169.0756(3.5), 163.0764(0.9), 160.0537(0.7), 137.0614(2.2), 131.0511(2.0)
1-(4-hydroxyphenyl)-7-phenyl-(6E)-6-hepten-3-ol	C_19_H_22_O_2_	PPN	[M + Cl] −	317.1345	131.0808 (0.3)
Sterculynic acid	C_19_H_30_O_2_	FAT	[M + H − H_2_O] +	273.2235	273.2220(60.9), 255.2121(53.2), 173.1339(6.3), 163.0616(4.3), 161.1336(28.8), 147.1183(8.0)
Kaempferol 3-O-arabinoside	C_20_H_18_O_10_	FLV	[M + H − 2H_2_O] +	383.0783	325.0730(4.0), 299.0568(7.3), 165.0197(0.5)
all-trans-3,4-Didehydroretinoate	C_20_H_26_O_2_	PRN	[M + H] +	281.1929	181.1024(21.3), 165.0731(19.1), 157.1027(23.0), 155.0870(37.3), 145.1027(27.6), 128.0636(66.9)
Cinncassiol C	C_20_H_28_O_7_	TER	[M + H − H_2_O] +	363.1781	332.1368(0.6), 314.1253(0.5), 222.1141(0.6), 136.0677(2.1), 135.0456(0.2), 119.0495(0.8)
Isopimaric acid	C_20_H_30_O_2_	TER	[M + H − 2H_2_O] +	285.2239	284.2974(0.7), 257.2282(2.8)
2′-Hydroxyisoorientin	C_21_H_20_O_12_	FLV	[M + H] +	465.1051	303.0512 (7.4)
5,7,3′-trihydroxy-3,5′-dimethoxy-2′-(3′-methylbut-2-enyl)flavone	C_22_H_22_O_7_	FLV	[M + H] +	399.1472	381.1379 (0.9)
Vestitone 7-glucoside	C_22_H_26_O_9_	PPN	[M + ACN + H] +	417.1577	221.0831 (2.6)
6-O-Acetylaustroinulin	C_22_H_36_O_4_	TER	[M + ACN + Na] +	787.5307	733.4879 (7.0)
xi-8-Acetonyldihydrosanguinarine	C_23_H_19_NO_5_	AK	[M + H − H_2_O] +	372.1245	344.1288(0.8), 149.0352(0.3)
Quercetin 3-(6″-malonyl-glucoside)	C_24_H_22_O_15_	FLV	[M + H] +	551.1061	303.0514 (23.3)
12′-apo-β-carotenal	C_25_H_34_O	TER	[2M + FA − H] −	745.5259	685.5227(231.2), 539.4294(47.0)
Kaempferol 3-xylosylglucoside	C_26_H_28_O_15_	FLV	[M + H] +	581.1525	341.2486(1.6), 287.0557(49.5), 153.0195(2.8), 131.0512(2.3)
11′-Carboxy-α-tocopherol	C_26_H_42_O_4_	TPHE	[M + H] +	419.3222	177.1023 (0.5)
β-tocopherol	C_28_H_48_O_2_	TPHE	[2M − H] -	831.7267	417.6959(90.2)
Amarogentin	C_29_H_30_O_13_	GC	[M + NH4] +	604.2051	325.073(4.0), 299.0568(7.3), 165.0197(0.5)
Rhamnazin 3-rutinoside	C_29_H_34_O_16_	FLV	[M + 2Na − H] +	683.1485	303.0514 (23.3)
Myrciacitrin V	C_30_H_30_O_13_	FLV	[M + ACN + H] +	640.2098	151.0407 (5.4)
Bryononic acid	C_30_H_46_O_3_	CBN	[M + ACN + Na] +	518.3645	358.1972(0.5), 342.2300(1.5), 320.2464(1.8), 222.1338(0.3), 196.1848(0.3)
3,7-Dihydroxy-25-methoxycucurbita-5,23-dien-19-al	C_31_H_50_O_4_	STR	[M + Cl] −	521.3404	485.7277(21.0)
Capsianoside I	C_32_H_52_O_14_	TER	[M + Na] +	683.3298	683.3291(4.4), 365.1088(1.6), 363.0929(1.6), 271.2444(7.5)
Diosgenin 3-O-beta-d-glucoside	C_33_H_52_O_8_	TER	[M + H] +	577.3759	468.2101(67.0), 441.1756(9.8), 415.3230(23.2), 397.3135(4.5), 397.1857(3.8), 271.0622(4.8)
Kidjoranin-3-O-β-digitoxopyranoside	C_36_H_48_O_10_	SPNS	[M − H_2_O − H] −	621.3029	621.3011(167.6), 579.2889(8.2), 285.1144(46.0), 255.0975(5.6)
Feruloyl-β-sitosterol	C_39_H_58_O_4_	TER	[2M + Hac − H] −	1239.9012	1239.893(28.0), 887.5754(11.0)
Ubiquinol-6	C_39_H_60_O_4_	PRN	[M + Na] +	615.4544	394.3743(20.0), 322.2779(2.0), 310.3341(3.8), 134.1078(2.0)
Fistuloside A	C_39_H_62_O_13_	SPNS	[M + H] +	739.4309	577.3766(32.5), 468.2101(67.0), 441.1756(9.8), 415.3230(23.2), 397.3135 (4.5),271.0622(4.8)
Nigroxanthin	C_40_H_54_O_2_	TER	[M + 2Na − H] +	611.3841	467.2684(1.0), 449.3285(2.3), 305.2134(4.5), 287.2032(2.6), 269.1927(1.9)
Ursolic acid 3-[glucosyl-(1->4)-xyloside]	C_41_H_66_O_12_	TER	[M + Na] +	773.4399	773.4389(4.5), 686.3793(2.1), 611.3844(5.0), 449.3285(2.3), 305.2134(4.5), 287.2032(2.6)
Melilotoside B	C_41_H_68_O_12_	TER	[M + H] +	753.4203	267.1773 (0.4)
Licoricesaponin C2	C_42_H_62_O_15_	TER	[M + Na] +	829.3952	829.3948(32.4), 723.3561(3.4),624.3786(4.6),310.1940(1.3), 250.1564(8.8), 146.0618(1.3)
Tuberoside L	C_51_H_84_O_23_	SPNS	[M + H] +	1065.5627	670.3848(1.4), 611.3847(1.6), 449.3291(1.6), 432.3226(0.5)
Yamogenintetroside B	C_52_H_86_O_22_	SPNS	[M + 2Na − H] +	1107.5319	854.4602(7.0), 762.4256(11.1), 559.4917(1.7), 541.4820(2.5), 426.3396(5.1), 309.1197(1.9)
Oleanolic acid 3-O-[O-β-d-glucopyranosyl-(1->4)-O-β-d-glucopyranosyl-(1->3)-O-α-l-rhamnopyranosyl-(1->2)-α-l-arabinopyranoside]	C_53_H_86_O_21_	TER	[M + Na] +	1081.5464	773.4401(1.3), 611.3846(3.4), 449.3298(3.2), 153.0195(6.5)
Tragopogonsaponin F	C_56_H_80_O_21_	SPNS	[M + CH_3_OH + H] +	1121.5515	786.4322 (43.4)
Trigofoenoside G	C_56_H_92_O_27_	SPNS	[M + H] +	1197.5995	1197.5994(31.3), 829.3948(32.4), 723.3561(3.4), 624.3786(4.6), 338.1889(1.5), 250.1564(8.8)
Hovenoside D	C_57_H_92_O_26_	TER	[M + CH_3_OH + H] +	1225.6028	1210.6307(124.9), 1064.5708(55.2), 870.4542(25.2), 442.3347(11.2), 325.1173(14.4), 301.0726(19.0)
Capsicosin	C_57_H_94_O_29_	TER	[M + H] +	1243.6144	595.3883(18.9), 433.3333(18.6), 415.3237(9.2), 289.2185(18.0), 271.2091(10.8), 161.1340(12.7)
Eleutheroside L	C_59_H_96_O_25_	SPNS	[M + Na] +	1227.6129	932.493(12.4), 399.3288(8.4), 285.2599(2.2)
β-l-arabinose 1-phosphate(2-)	C_5_H_9_O_8_P-2	GC	[M + Cl] −	262.9693	262.9688(2.6), 218.9505(4.6)
Capsicoside A	C_63_H_106_O_35_	SPNS	[M + H − 2H_2_O] +	1387.6618	901.4882(5.1), 739.4320(24.5), 577.3766(32.5), 468.2101(67.0), 441.1756(9.8), 415.3230(23.2)
Matesaponin 5	C_65_H_10_6O_31_	SPNS	[M + Na] +	1405.6713	757.4389(195.6), 595.3839(200.6), 451.2716(56.9), 289.2162(151.2), 271.2075(66.5), 253.1970(56.8)
Pyridoxamine	C_8_H_12_N_2_O_2_	PYR	[M+H−H_2_O] +	151.0872	135.0247 (0.3)
3-[3,4-Dihydroxy-2-(hydroxymethyl)-1-pyrrolidinyl]propanamide	C_8_H_16_N_2_O_4_	AK	[M + H − H_2_O] +	187.1093	175.1117(1.3), 155.0443(0.2), 116.0711(1.3), 112.0767(0.5), 109.0296(0.9)
2,4-Nonadienal	C_9_H_14_O	CBN	[M + K] +	177.0683	169.1144(11.7), 157.1133(15.3), 155.0982(17.7), 153.0822(13.1), 142.0889(25.6), 128.0724(24.6)

AK: Alkaloids; BZD: benzoyl derivate; CAPS: capsaicinoids; CBN: carbonyl derivate; FAT: fatty acids; FLV: flavonoids; GC: glycoside compounds; Jasm: jasmones; PPN: phenylpropanoids; PYR: pyrazines; SPNS: saponins; TER: terpenoids; TPHE: tocopherols. Relative abundance values of fragments ions are in brackets.

## Supplementary Materials

The following are available online at https://www.mdpi.com/2218-1989/9/10/206/s1, Table S1: Putative identification.xlsx.
